# Reports of Encephalopathy Among Children with Influenza-Associated Mortality — United States, 2010–11 Through 2024–25 Influenza Seasons

**DOI:** 10.15585/mmwr.mm7406a3

**Published:** 2025-02-27

**Authors:** Amara Fazal, Katie Reinhart, Stacy Huang, Krista Kniss, Samantha M. Olson, Vivien G. Dugan, Sascha Ellington, Alicia P. Budd, Carrie Reed, Timothy M. Uyeki, Shikha Garg

**Affiliations:** 1 Influenza Division, National Center for Immunization and Respiratory Diseases, CDC.

**Summary**

**What is already known about this topic?**

Influenza-associated encephalopathy or encephalitis (IAE), including acute necrotizing encephalopathy (ANE), is a rare and potentially fatal complication of influenza. No national IAE surveillance exists.

**What is added by this report?**

During late January 2025, CDC received anecdotal reports of critically ill children with IAE, including deaths with ANE. Data from the Influenza-Associated Pediatric Mortality Surveillance System was investigated and revealed the median proportion of pediatric influenza deaths with IAE during the 2010–11 through 2024–25 influenza seasons was 9%. IAE was identified in 13% (nine of 68) of deaths during the 2024–25 influenza season (through February 8, 2025), including four with ANE.

**What are the implications for public health practice?**

It is not known whether cases observed in the 2024–25 season vary from expected numbers. Clinicians should consider IAE in children with influenza and abnormal neurologic signs or symptoms. Influenza vaccination is recommended for all persons aged ≥6 months while influenza viruses are circulating.

## Abstract

In late January 2025, CDC received anecdotal reports of children with influenza-associated acute necrotizing encephalopathy (ANE), a severe form of influenza-associated encephalopathy or encephalitis (IAE), including several fatal cases. In response, CDC examined trends in the proportions of cases with IAE among influenza-associated pediatric deaths reported during the 2010–11 through 2024–25 influenza seasons, including demographic and clinical characteristics of identified cases. CDC contacted state health departments to ascertain whether any pediatric influenza-associated deaths with IAE reported this season also had a diagnosis of ANE. Among 1,840 pediatric influenza-associated deaths during the 2010–11 through 2024–25 influenza seasons, 166 (9%) had IAE, ranging from 0% (2020–21 season) to 14% (2011–12 season); preliminary data for the 2024–25 season (through February 8, 2025) indicate that nine of 68 (13%) had IAE. Across seasons, the median age of patients with fatal IAE was 6 years; 54% had no underlying medical conditions, and only 20% had received influenza vaccination. Because no dedicated national surveillance for IAE or ANE exists, it is unknown if the numbers of cases this season vary from expected numbers. Health care providers should consider IAE in children with acute febrile illness and neurologic signs or symptoms lasting >24 hours. Evaluation should include testing for influenza and other viruses and neuroimaging; clinical management should include early antiviral treatment for suspected or confirmed influenza and supportive critical care management as needed. Influenza vaccination is recommended for all eligible persons aged ≥6 months as long as influenza viruses are circulating.

## Introduction

Children of all ages, and especially those aged <5 years with certain underlying medical conditions, can experience severe or fatal complications associated with influenza virus infection (*1*), including pneumonia, myocarditis, pericarditis, and neurologic complications.* Influenza-associated encephalopathy or encephalitis (IAE) comprises a spectrum of neurologic syndromes that are triggered by influenza virus infection of the respiratory tract, resulting in a dysregulated host inflammatory response, leading to varying degrees of brain dysfunction, inflammation, or both (*2*,*3*). Acute necrotizing encephalopathy (ANE) is one of the most severe forms of encephalopathy and is a known complication of infection with influenza and other viruses (including SARS-CoV-2 and human herpesvirus 6) (*3*). The diagnosis of ANE is based on characteristic symmetric lesions affecting the bilateral thalami and other parts of the brain detected by head computed tomography or magnetic resonance imaging, in a child with febrile illness preceding or concurrent with the onset of neurologic signs or symptoms and rapid neurologic decline. In late January 2025, public health partners alerted CDC with anecdotal reports of pediatric hospitalizations with IAE, including several fatal cases with ANE. Data from CDC’s U.S. Influenza-Associated Pediatric Mortality Surveillance System were reviewed to further investigate.

## Methods

### Data Source

The Influenza-Associated Pediatric Mortality Surveillance System^†^ is a national surveillance system that collects data on all identified influenza-associated pediatric deaths, which have been nationally notifiable in the United States since 2004. An influenza-associated pediatric death is defined as the death of a person aged <18 years who has laboratory-confirmed influenza and a clinically compatible illness, with no period of complete recovery between illness and death.

### Identification of Cases of IAE and ANE

State and local health departments report influenza-associated pediatric deaths to CDC and use a standardized case report form to collect information on demographic characteristics, influenza laboratory results, underlying medical conditions, clinical course, acute complications, and influenza vaccination status.^§^ Trends in the proportions of cases of encephalopathy or encephalitis (included as a checkbox in the acute complications section of the case report form) among influenza-associated pediatric deaths reported to CDC during the 2010–11 through 2024–25 seasons were examined, including the demographic and clinical characteristics of identified cases. For this analysis, a case of IAE was defined as the marking of the encephalopathy or encephalitis checkbox on the case report form. During the 2024–25 season, for any ANE cases that had not already been reported to CDC, state health departments were contacted to ascertain whether any reported pediatric influenza-associated deaths with IAE identified through routine surveillance also had a diagnosis of ANE, because information regarding ANE is not specifically collected on the case report form. SAS software (version 9.4; SAS Institute) was used to conduct all analyses. This activity was reviewed by CDC, deemed not research, and was conducted consistent with applicable federal law and CDC policy.^¶^

## Results

### IAE Cases Reported During the 2010–11 Through 2024–25 U.S. Influenza Seasons

Among 1,840 pediatric influenza-associated deaths reported to CDC during the 2010–11 through 2024–25 influenza seasons, 166 (9%) had IAE, ranging from 0% (2020–21 season) to 14% (2011–12 season); preliminary data for the 2024–25 season (reported October 1, 2024–February 8, 2025) indicate that nine of 68 (13%) deaths had IAE (Table 1). By age group, IAE prevalence was highest among children aged 2–4 years (34 [10%] of 334 total fatal cases) and lowest among infants aged <6 months (eight [5%] of 148 fatal cases). 

**TABLE 1:** Pediatric influenza-associated deaths (N = 1,840) and deaths with influenza-associated encephalopathy or encephalitis (n = 166), by influenza season and age group — Influenza-Associated Pediatric Mortality Surveillance System, United States, 2010–11 through 2024–25* influenza seasons

Characteristic	No. of pediatric influenza-associated deaths^†^	No. of influenza-associated deaths with IAE diagnosis (row %)
**Influenza season**		
2010–11	124	12 (10)
2011–12	37	5 (14)
2012–13	171	10 (6)
2013–14	111	4 (4)
2014–15	148	15 (10)
2015–16	95	8 (8)
2016–17	110	9 (8)
2017–18	188	16 (9)
2018–19	145	13 (9)
2019–20	199	20 (10)
2020–21	1^§^	0 (—)
2021–22	49	5 (10)
2022–23	187	17 (9)
2023–24	207	23 (11)
2024–25*	68	9 (13)
**Age group**		
0–6 mos	148	8 (5)
6–23 mos	311	28 (9)
2–4 yrs	334	34 (10)
5–11 yrs	635	62 (10)
12–17 yrs	412	34 (8)
**Total 0–17 yrs**	**1,840**	**166 (9)**

**Abbreviation**: IAE = influenza-associated encephalopathy or encephalitis.

* Preliminary data through February 8, 2025.

**^†^** Data available at https://gis.cdc.gov/grasp/fluview/pedfludeath.html

^§^ Influenza activity was historically low during the COVID-19 pandemic. https://www.cdc.gov/mmwr/volumes/69/wr/mm6937a6.htm

### Characteristics of IAE Cases

Among the 166 fatal pediatric influenza-associated cases with IAE, the median age was 6 years (IQR = 2.5–10.5 years), 52% were female, and 40% were non-Hispanic White (Table 2). Overall, 119 (72%) patients had influenza A, and 46 (28%) had influenza B virus infection. Among 73 influenza A cases with available subtype, 41 (56%) had A(H1N1)pdm09 and 32 (44%) had A(H3N2). No underlying medical conditions were reported for more than one half of fatal cases (89; 54%); 32 patients (20%) had received ≥1 dose of current season influenza vaccine >2 weeks before illness onset, and 121 (73%) received influenza antiviral treatment. Overall, 155 (93%) patients required mechanical ventilation; other documented acute complications included acute respiratory distress syndrome (57; 34%), pneumonia (54; 33%), and sepsis (47; 28%). Among all fatal cases, 159 (96%) patients died during hospitalization, 2% died in the emergency department, and 2% died outside the hospital setting.

**TABLE 2:** Characteristics of children with influenza-associated mortality and influenza-associated encephalopathy or encephalitis (N = 166) — Influenza-Associated Pediatric Mortality Surveillance System, United States, 2010–11 through 2024–25* influenza seasons

Characteristic (no. with available information)	No. with IAE (%)
**Total no. with IAE**	**166 (100)**
Median age, yrs (IQR)	6.0 (2.5–10.5)
**Sex (166)**	
Male	80 (48)
Female	86 (52)
**Race and ethnicity (166)^†^**	
American Indian or Alaska Native	2 (1)
Asian or Pacific Islander	20 (12)
Black or African American	22 (13)
White	66 (40)
Hispanic or Latino	47 (28)
Unknown/Missing	9 (5)
**Influenza type/Subtype (166)**	
Influenza A	119 (72)
Known A subtype	73 (61)
A(H1N1)	41/73 (56)
A(H3N2)	32/73 (44)
Unknown A subtype	46 (39)
Influenza B	46 (28)
A/B not distinguished	1 (<1)
**≥1 underlying medical condition (166)**	
Yes	74 (45)
No	89 (54)
Unknown/Missing	3 (2)
**Received current season influenza vaccine >14 days before illness onset (158)** ^§^	
Yes	32 (20)
No	96 (61)
Unknown/Missing	30 (19)
**Receipt of antiviral treatment (166)**	
Yes	121 (73)
No	45 (27)
**Required mechanical ventilation (166)**	
Yes	155 (93)
No	6 (4)
Unknown	5 (3)
**Other acute complications (166)** ^¶^	
Pneumonia	54 (33)
Sepsis	47 (28)
ARDS	57 (34)
**Location of death (166)**	
Outside of hospital	3 (2)
Emergency department	4 (2)
Hospital	159 (96)

**Abbreviations**: ARDS = acute respiratory distress syndrome; IAE = influenza-associated encephalopathy or encephalitis.

* Data for the 2024-25 influenza season are preliminary through February 8, 2025.

**^†^** Persons of Hispanic or Latino (Hispanic) ethnicity might be of any race but are categorized as Hispanic; all racial groups listed here are non-Hispanic.

^§^ Eight children are excluded from this denominator because they were aged <6 months and not eligible for influenza vaccination.

^¶^ Some children had multiple complications, thus numbers reported are not mutually exclusive.

### IAE and ANE Cases During the 2024–25 Influenza Season

Nine pediatric influenza-associated deaths with IAE were reported to CDC during the 2024–25 season through February 8, 2025; four had documented ANE (two ANE deaths were proactively reported to CDC by state health departments, and two additional deaths were identified during CDC outreach to states). All four ANE deaths were aged <5 years; one child had underlying medical conditions, and all four had laboratory-confirmed influenza A(H1N1)pdm09. Two children with ANE had received influenza vaccination >2 weeks before illness onset; the others had not received influenza vaccination during the 2024–25 influenza season. Two children with ANE received oseltamivir treatment, two experienced seizures during hospitalization, and all four received mechanical ventilation.

## Discussion

CDC has received recent anecdotal reports of critically ill children with influenza-associated ANE, including several deaths, during the 2024–25 influenza season. CDC does not systematically collect data on either influenza-associated ANE or IAE cases; however, national pediatric influenza-associated mortality surveillance during the 2024–25 season (through February 8, 2025) detected IAE in 13% of cases; four of the children had a diagnosis of ANE. Because there is no dedicated U.S. surveillance for IAE (including ANE) among children, it is currently not known whether these reported cases vary from expected numbers. Enhanced surveillance to systematically identify and report pediatric IAE cases, including ANE, in the United States during the remainder of the 2024–25 season would improve understanding of the incidence of this influenza complication and the frequency of severe outcomes, including long-term neurologic sequelae or death. Thus, on February 24, 2025, CDC posted a national call for possible cases of pediatric IAE identified during this influenza season (October 1, 2024, through May 30, 2025) on the Epidemic Information Exchange (EPI-X). CDC can be contacted at severeflu@cdc.gov to begin the case reporting process; no case information, including protected health information, should be communicated over email. Additional information can be found at https://epix2.cdc.gov/v2/Reports/Display.aspx?id=541771. 

IAE is not notifiable in the United States; however, in Japan, where encephalopathy and encephalitis due to an infection is notifiable, the number of cases among persons of all ages during 2010–2015 ranged from 64 to 105 per season. Seventy-four percent of cases were in persons aged <18 years, and IAE case-fatality among persons aged <18 years was 8% (*4*).

Three major IAE syndromes have been described. ANE is the most severe form and is associated with high rates of long-term neurologic sequalae and death, followed by acute encephalopathy with biphasic seizures and late reduced diffusion (a finding on magnetic resonance imaging that typically signifies tissue damage or abnormality), and clinically mild encephalitis or encephalopathy with a reversible splenial lesion (*2*). Other less commonly reported IAE syndromes include acute encephalopathy with refractory partial seizures and posterior reversible encephalopathy syndrome (*2*). Although no standardized IAE case definition currently exists, consensus definitions for infection-triggered encephalopathy syndromes have recently been published (*3*). Criteria for diagnosis of ANE, including influenza-associated ANE, are well characterized and include febrile illness preceding or concurrent with the onset of neurologic signs or symptoms, rapid neurologic decline, and neuroimaging demonstrating symmetric lesions affecting the bilateral thalami and other parts of the brain (*3*).

The following features that have been described in pediatric cases of IAE can be useful for surveillance purposes or clinical diagnosis, in conjunction with clinical judgment: 1) age <18 years; 2) laboratory-confirmed influenza virus infection; 3) diagnosis of encephalopathy or encephalitis or neurologic signs or symptoms, including seizures, altered mental status, delirium, decreased level of consciousness, lethargy, hallucinations, or personality changes lasting >24 hours; and 4) neuroimaging abnormalities (not always present) such as brain edema, inflammation, or brain lesions, or electroencephalographic abnormalities; in the absence of other known causes of disease (*3*–*5*). 

Progression to severe neurologic impairment and death from IAE can occur rapidly after onset of influenza symptoms; thus, prompt recognition and intervention are crucial, including neurocritical supportive care for patients with increased intracranial pressure and management of multiorgan failure. Early initiation of antiviral treatment is recommended for children at increased risk for influenza-associated complications, although whether antiviral treatment is beneficial for management of IAE is unknown. Notably, one study reported that oseltamivir treatment was associated with a reduced risk for neuropsychiatric events among patients with influenza (*6*). Although there are currently no international evidence-based guidelines for standardized clinical management of patients with IAE, high-dose pulse methylprednisolone, plasma exchange, therapeutic hypothermia, and immune therapy such as gamma globulin, anakinra (an interleukin-1 receptor antagonist), and tocilizumab (an interleukin-6 receptor blocker) have been used (*6*–*8*). In one study, use of the nonsteroidal anti-inflammatory drug diclofenac sodium (but not acetaminophen) was associated with increased mortality in IAE cases (*5*). Additional studies are needed to identify optimal strategies for clinical management of IAE.

### Limitations

The findings in this report are subject to at least four limitations. First, diagnoses of encephalopathy or encephalitis are currently captured in checkboxes on the pediatric influenza-associated mortality case report form; these data might have over- or underestimated the true prevalence of IAE. Second, data on IAE prevalence among pediatric influenza-associated deaths during the 2024–25 season are preliminary and based on small numbers; results might change as additional data become available. Third, the prevalence of IAE among pediatric influenza-associated deaths is likely not representative of overall pediatric IAE prevalence in the United States, especially less severe IAE cases. Finally, given the lack of established surveillance for IAE in the United States, it was not possible to ascertain whether anecdotal reports of pediatric IAE (including influenza-associated ANE) hospitalizations and deaths during the current influenza season are within or above expected ranges.

### Implications for Public Health Practice

Health care providers should consider IAE in children with febrile illness and clinically compatible neurologic signs or symptoms, including but not limited to seizures, altered mental status, delirium, decreased level of consciousness, lethargy, hallucinations, or personality changes lasting >24 hours. Influenza-associated ANE should be considered in children with signs or symptoms of IAE, as well as rapid neurologic decline and neuroimaging demonstrating symmetric lesions affecting the bilateral thalami and other parts of the brain. Comprehensive assessment and management should include testing for influenza and other viruses, neuroimaging, early initiation of antiviral treatment if influenza is confirmed or suspected (i.e., providers should not wait for laboratory confirmation of influenza before initiating antiviral treatment), and supportive critical care management as needed for patients with IAE. Use of standardized criteria by health care providers for IAE case identification and establishment of a mechanism for public health reporting will improve understanding of the incidence and impact of this serious influenza complication. CDC has posted a national call for possible pediatric IAE cases identified during this influenza season on EPI-X and can be contacted at severeflu@cdc.gov. 

Influenza vaccination is an important tool for preventing influenza and its associated complications (*1*). U.S. influenza activity is currently elevated, and influenza viruses could continue circulating into the spring; thus, health care providers should provide a strong recommendation for influenza vaccination for all eligible persons aged ≥6 months who have not yet been vaccinated this season to prevent influenza illness and its associated severe and potentially fatal complications (*9*).

## Footnotes

*
https://www.idsociety.org/practice-guideline/influenza/

†
https://www.cdc.gov/fluview/overview/index.html#cdc_generic_section_5-mortality-surveillance

§ Health departments verify vaccination status by reviewing medical records, contacting health care providers, or checking state vaccine registries.

¶ 45 C.F.R. part 46.102(l)(2), 21 C.F.R. part 56; 42 U.S.C. Sect. 241(d); 5 U.S.C. Sect. 552a; 44 U.S.C. Sect. 3501 et seq.
